# Synergistic Role between p53 and JWA: Prognostic and Predictive Biomarkers in Gastric Cancer

**DOI:** 10.1371/journal.pone.0052348

**Published:** 2012-12-21

**Authors:** Xin Liu, Shouyu Wang, Xiaowei Xia, Yansu Chen, Yan Zhou, Xuming Wu, Jianbing Zhang, Song He, Yongfei Tan, Fulin Qiang, Oluf Dimitri Røe, Gang Li, Jianwei Zhou

**Affiliations:** 1 Department of Molecular Cell Biology and Toxicology, Jiangsu Key Lab of Cancer Biomarkers, Prevention & Treatment, Cancer Center, School of Public Health, Nanjing Medical University, Nanjing, People’s Republic of China; 2 Department of Oncology, Yixing Hospital, Yixing, Jiangsu Province, People’s Republic of China; 3 Department of Pathology, Nantong Cancer Hospital, Nantong, Jiangsu Province, People’s Republic of China; 4 Department of Cancer Research and Molecular Medicine, Norwegian University of Science and Technology, Trondheim, Norway; 5 Department of Dermatology and Skin Science, Jack Bell Research Centre, Vancouver Coastal Health Research Institute, University of British Columbia, Vancouver, British Columbia, Canada; Vanderbilt University Medical Center, United States of America

## Abstract

Expression of p53 appears to be correlated to prognosis in patients with malignancy, but its role in gastric carcinoma has remained controversial. Recently we reported that JWA, an ADP-ribosylation-like factor 6 interacting protein 5 (ARL6ip5), was both prognostic for overall survival and predictive for platinum-based treatment of gastric cancer. In this study, we aimed to investigate p53 expression as a prognostic and predictive marker in resectable gastric cancer, alone and in combination with JWA. Expression of p53 was examined in three large patient cohorts (total n = 1155) of gastric cancer. High expression of p53 was significantly correlated with unfavorable clinicopathologic parameters and decreased overall patient survival. Furthermore, patients with high p53 expression in tumors acquired remarkable survival benefit from adjuvant first-line platinum-based-chemotherapy. The synergy between p53 and JWA in predicting patient outcome was demonstrated, while no significantly elevated predictive value concerning chemotherapy was observed. Thus, p53 expression is a potent prognostic and predictive factor for resectable gastric cancer with adjuvant platinum-based chemotherapy. A combined effect of p53 with JWA as efficient prognostic indicators was found for the first time.

## Introduction

Gastric cancer affects about one million people a year, being the second leading cause of cancer-related mortality worldwide with an overall five-year survival rate of less than 30% [1]. The poor outcome has remained basically unchanged over the last decades in spite of improvements in surgical, chemo- and radiotherapy [2]. Striking differences in prognosis among patients after standard surgery exist, indicating specific biomarkers are urgently needed [3]. Moreover, selection of patients who will benefit from chemotherapy is a major issue as a large fraction is unnessesarily treated and only get severe side effects [4]. Thus, efficient molecular markers are urgently needed to avoid over- or undertreatment.

The p53 protein (encoded by the human gene *TP53*) is possibly the best known of all tumor suppressors, and characterized by the ability to induce cell cycle arrest, DNA repair, senescence, and apoptosis [5]. Mutation or functional inactivation of p53 is an almost universal feature of human cancer, playing a crucial role in tumorigenesis since mutant p53 may acquire new oncogenic properties [6]–[9]. Furthermore, unlike wild-type p53 protein that is degraded rapidly, mutant forms have a prolonged half-life, which favor intranuclear storage, becoming detectable immunohistochemically [10]–[12]. The accumulation of p53 protein in the case of gastric cancer has been linked to prognosis and prediction of treatment [13], [14]. However, its related role and mechanism hitherto remain controversial [15], [16].

Our recent findings implicated that JWA, also known as ARL6ip5, similar with p53 in some aspects, is essential for cell survival and efficient DNA repair after oxidative DNA damage, as well as chemically induced cancer cell apoptosis [17], [18]. Moreover, JWA and XRCC1, another DNA repair protein, may be candidate prognostic and predictive biomarkers for patients with gastric cancer [19]. Additionally, SNPs of the *JWA* gene are associated with increased predisposition to gastric carcinoma in a Chinese population [20]. Thus, we are considerably interested if JWA would work as a cooperator with p53 to improve predictive potency in gastric cancer.

Herein, we aimed to elucidate the translational significance and identify the expression patterns of p53 in three large independent cohorts of gastric cancer patients and to examine the possible prognostic and predictive role of this marker. More intriguingly, a hypothesis would be validated on whether p53 and JWA could be combined as a novel predictor with more accuracy in survival evaluation.

## Materials and Methods

### Patients and Samples

Three independent retrospective patient cohorts were studied. The training cohort and testing cohort were collected in Nantong Cancer Hospital, Nantong City, in the east part of Jiangsu Province and the validation cohort was recruited in Yixing People’s Hospital, Yixing City, in the south part of Jiangsu Province, China. The tissues were obtained from the respective pathology divisions. Inclusion criteria were gastric carcinoma treated with radical gastrectomy with or without adjuvant chemotherapy. Exclusion criteria were patients with previous gastric cancer or active non-gastric cancer. Also, those who received pre-surgical chemo- or radiation therapy were excluded. Written informed consent was obtained from each patient prior to tissue acquisition and before surgery was carried out. Institutional approval was acquired from the Ethical Review Board of Nanjing Medical University prior to this study.

### Patients Treated with Surgery Alone

The training cohort included 103 patients who only underwent radical gastrectomy at Nantong Cancer Hospital from 1^st^ May 1990 to 1^st^ June 1995. However, 20 samples were omitted because of missing data and one sample was lost during antigen retrieval or without tumor cells present in the core, so 82 paired patient tissues were finally evaluated for p53 expression.

The testing cohort consisted of all 640 surgical cases from the Nantong Cancer Hospital from 1^st^ December 2000 to 1^st^ April 2005 and the validation cohort included all 1022 surgical cases in Yixing People’s Hospital from 1^st^ January 1999 to 31^st^ December 2006. These patients were treated with surgery only or with postoperative adjuvant chemotherapy (for details, see Fig. S1). The distributions of demographic characteristics and the selected clinicopathologic variables of patients between the two districts (Nantong and Yixing) were described previously [19]. Due to missing survival data, only 578 and 998 of these patients were included. Furthermore, due to lost cores or insufficient tumor cells in the tissue, 485 and 588 tumor cores were analyzed for p53 expression in the testing and validation cohort, respectively.

### Patients Treated with Adjuvant Chemotherapy

In the training cohort, none of the patients received any form of adjuvant therapy. Of the 485 patients used for analysis in the testing cohort, 111 patients (22.9%) were treated with adjuvant chemotherapy after curative resection. The regimens included combined chemotherapy with fluorouracil, leucovorin, and oxaliplatin (FLO) (6 cases); monotherapy with mitomycin C (46 cases); fluorouracil derivatives (47 cases); and other treatments (12 cases). Of the 588 patients in the validation cohort, the regimen for 223 patients (37.9%) with postoperative chemotherapy included combined chemotherapy with FLO (87 cases); combined chemotherapy with fluorouracil, leucovorin, and platinol (FLP) (79 cases); combined chemotherapy with fluorouracil and paclitaxel (FP) (11 cases); combined chemotherapy with etoposide, leucovorin, fluorouracil and platinol (ELFP) (28 cases); and other treatments (18cases). Regarding the resectable gastric cancer patients with chemotherapy, the distributions of demographic characteristics and the selected clinicopathologic variables of patients between FLO group and FLP group were similar (all of *P*>.05), except histological type (*P* = .001, Table S1). In addition, 7 pathologically confirmed gastric cancer and respective non-cancerous fresh frozen gastric mucosa tissues from recent patients in Nantong Cancer Hospital were obtained for Western blot analysis.

Overall survival (OS) was the primary end-point of this analysis. Survival time was ascertained from the date of surgery to the date of death or to the last follow-up. Date of death for each case was obtained from patient records or patients’ families through follow-up telephone calls and further double-verified by local civil affairs department and public security department. Detailed clinicopathologic information was obtained. Lauren’s criteria were used to classify the tumors into intestinal type or diffuse type [21] and staged according to the Tumor, Node, Metastasis (TNM) guidelines [22].

### Construction of Tissue Microarray (TMA) and Immunohistochemistry

Paraffin-embedded archived tissue material of tumor and surrounding normal gastric tissue was used for TMA construction. TMAs were prepared as previously published [19]. In brief, duplicate 1.0 mm diameter cores of tissue from each sample were punched from paraffin tumor block and corresponding non-tumoral tissues in the training cohort or from cores of primary tumor biopsies in testing and validation cohorts. As a tissue control, the biopsies of normal gastric epithelium tissues were inserted in the four angles and the center of each slide.

A standard protocol was used for the immunostaining of the TMAs. The detailed process was described earlier [19]. IHC was performed using DO7 monoclonal antibody (1∶80; Dako, Carpinteria, CA), which detects the wild-type and mutant forms of the p53-protein, together with Elivision™ super HRP Kits (Maixin-Bio, Fujian, China), applying varying detection and antigen retrieval methods. The omission of the primary antibody served as negative control. The staining scores of the tissue controls in each microarray slide were pre-evaluated as a quality control of the immunostaining.

### Evaluation of Immunohistochemistry

At first, staining of p53 in the tissue were scored independently by two pathologists blinded to the clinical data, by applying a semi-quantitative immunoreactivity score (IRS) in the training cohort. The scoring criteria for IRS were reported elsewhere [23]. The intensity of immunostaining was shown in Fig. S2. The concordance for IRS staining score of p53 between the two pathologists was 74 (90%) in 82 tumors of the training set; and the few discrepancies were resolved by consensus using a multihead microscope. The variability in p53 staining was 4 (5%) in the duplicate cores of 82 tumors. These cases were stained by whole-slide IHC and further scored.

The optimum cutoff value of IRS is obtained by receiver-operator characteristic (ROC) analysis, and the area under the curve (AUC) at different cutoff values of p53 IRS for 1, 3 and 5 years of overall survival time was calculated. The optimal value of cutoff points of p53 IRS in Nantong district cohort (combined training cohort and testing cohort) was 4 due to the predictive value of this cutoff point for death was the best (Fig. S3). Under these conditions, samples with IRS 0–4 and IRS 5–12 was classified as low and high expression of p53 in tumors, respectively. After establishing the immunohistochemical assessment criteria in the Nantong district cohort, the expression of p53 in the Yixing district cohort (validation cohort) was scored by the same pathologists with the exactly same procedure.

### Western Blotting

Western blotting was carried out as previously described [19]. Monoclonal mouse anti-p53 antibody (1∶1000; Dako, Carpinteria, CA), and monoclonal mouse anti-β-actin antibody (1∶2000; Beyotime Biotechnology, Nantong, China) were used for the primary antibody. Immunoreactive bands were detected with a Phototope-HRP Western blot detection kit (Cell Signaling Technology Inc, Beverly, MA, USA). For densitometric analysis, p53 protein bands on the blots were measured by Image J software (version 1.44, Wayne Rasband, National Institutes of Health, USA), after normalization to the corresponding β-actin level.

### Statistical Analysis

The association between p53 expression and clinicopathologic parameters was evaluated by Fisher’s exact test. The differential expression of p53 in primary tumors and their corresponding non-tumors were assessed by the Wilcoxon test (grouped) and Spearman rank-order correlation (raw scores). The correlation between the expression of p53 and JWA was established by Spearman rank-order correlation (raw scores) and Fisher’s exact test (grouped). Probability of differences in OS as a function of time was ascertained by use of the Kaplan-Meier method, with a log-rank test for significance. Univariate or multivariate Cox regression analysis was performed to estimate the crude hazard ratios (HRs), adjusted HRs and their 95% confidence intervals (CIs), with adjustment for potential confounders. Then we analyzed the predictive value of the parameters using time-dependent ROC curve analysis for censored data and calculated AUC of the ROC curves. We evaluated the performances of different scores by plotting (t, AUC [t]) for different values of follow-up time (t). All the statistical analyses were performed by Statistical Analysis System software (version 9.1.3; SAS Institute, Cary, NC), STATA statistical software (version 10.1; StataCorp, College Station, TX), and R software (version 2.10.1; The R Foundation for Statistical Computing). A p-value of <.05 was deemed statistically significant.

## Results

### Increased p53 Expression in Gastric Cancer Versus Adjacent Normal Tissues

**Figure 1:**
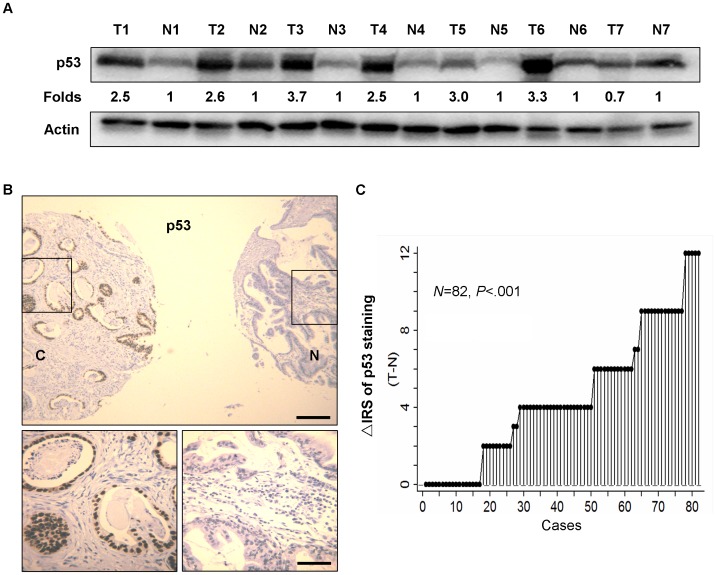
Correlation of p53 expression in primary tumors and corresponding non-tumors in gastric cancer patients. (A) p53 protein levels in 7 cancer tissues and paired non-cancerous normal tissues of gastric cancer patients were analyzed by Western blotting. The level of each protein was normalized against β-actin, and the protein levels in cancer tissues indicated as a ratio to paired non-cancerous normal tissues. Note: N, non-cancerous normal tissue; T, Tumor tissue. (B) Representative immunohistochemical staining for p53 in TMA. T, gastric cancerous tissue; N, paired non-cancerous gastric tissue. Top panel: scale bar, 250 µm; bottom panel: scale bar, 50 µm. (C) The distribution of the difference of p53 staining (Δ IRS = IRS T-IRS N). *P* values were calculated with the Wilcoxon test. IRS, immunoreactivity score.

Seven pairs of human gastric cancer samples, including primary gastric cancer tissues and matched normal gastric mucosa were selected to test p53 protein expression by Western blot. Elevated expression of p53 occurred in 6 of 7 gastric tumors compared with the paired normal gastric mucosa (Fig. 1A). Immunohistochemical staining of gastric tissue microarray (TMA) was used to further confirm p53 expression in 82 gastric cancer patients in the training cohort. It was shown that p53 staining was mainly localized in the nuclei (Fig. 1B). The distribution of the differences of IRS for p53 expression in tumors and matched non-tumors was shown in Fig. 1C. Moreover, p53 expression was significantly increased in 65 of 82 (79.2%) of gastric cancers compared with the matched normal gastric tissues (*P*<.001, Wilcoxon test; Fig. 1C). In all three independent cohorts of patients treated only with surgery only, the expression of p53 was negatively correlated with JWA in the cancerous tissues (*P*<.001 for all correlations, Table 1).

**Table 1:** Correlation between expression levels of p53 and clinicopathologic features of the individuals in three cohorts of gastric cancers treated with surgery alone.

Variables	Training cohort (n = 82 cases)			Testing cohort (n = 374 cases)			Validation cohort (n = 365 cases)		
	Low (%)	High (%)	*p* a	Low (%)	High (%)	*p* a	Low (%)	High (%)	*p* a
All patients	46 (56.1)	36 (43.9)		232 (62.0)	142 (38.0)		147 (40.3)	218 (59.7)	
Age (years)			.272			.222			.197
≤65	34 (73.9)	31 (86.1)		143 (61.6)	97 (68.3)		58 (39.5)	102 (46.8)	
>65	12 (26.1)	5 (13.8)		89 (38.4)	45 (31.7)		89 (60.5)	116 (53.2)	
Gender			.800			.729			.254
Males	35 (76.1)	26 (72.2)		163 (70.3)	97 (68.3)		109 (74.2)	173 (79.4)	
Females	11 (23.9)	10 (27.8)		69 (29.7)	45 (32.7)		38 (25.8)	45 (20.6)	
Depth of invasion			.379			.002			.043
T1/T2	4 (8.7)	1 (2.8)		56 (24.1)	16 (11.3)		60 (40.8)	66 (30.3)	
T3/T4	42 (91.3)	35 (97.2)		176 (75.9)	126 (88.7)		87 (59.2)	152 (69.7)	
Lymph node metastasis			<.001			<.001			.021
N0	19 (41.3)	1 (2.8)		87 (37.5)	17 (12.0)		67 (45.6)	72 (33.0)	
N1/N2/N3	27 (58.7)	35 (97.2)		145 (62.5)	125 (88.0)		80 (54.4)	146 (67.0)	
Distant metastasis			.003			1.000			.173
M0	43 (93.5)	24 (66.7)		221 (95.3)	136 (95.8)		144 (98.0)	207 (95.0)	
M1	3 (6.5)	12 (33.3)		11 (4.7)	6 (4.2)		3 (2.0)	11 (5.0)	
TNM stage			<.001			<.001			.057
I	8 (17.4)	1 (2.8)		35 (15.1)	5 (3.5)		47 (32.0)	46 (21.1)	
II	15 (32.6)	3 (8.3)		62 (26.7)	19 (13.4)		35 (23.8)	46 (21.1)	
III	18 (39.1)	17 (47.2)		107 (46.1)	91 (64.1)		62 (42.2)	119 (54.6)	
IV	5 (10.9)	15 (41.7)		28 (12.1)	27 (19.0)		3 (2.0)	7 (3.2)	
Tumor diameter			.119			<.001			.334
≤5 cm	27 (58.7)	14 (38.9)		111 (47.8)	40 (28.2)		88 (59.9)	119 (54.6)	
>5 cm	19 (41.3)	22 (61.1)		121 (52.2)	102 (71.8)		59 (40.1)	99 (45.4)	
Histological type^b^			1.000			.003			.082
Intestinal	24 (52.2)	18 (50.0)		146 (62.9)	66 (46.5)		68 (47.2)	82 (37.8)	
Diffuse	22 (47.8)	18 (50.0)		86 (37.1)	76 (53.5)		76 (52.8)	135 (62.2)	
JWA expression			<.001			<.001			<.001
Low	17 (37.0)	33 (91.7)		32 (13.8)	134 (94.4)		40 (27.2)	145 (66.5)	
High	29 (63.0)	3 (8.3)		200 (86.2)	8 (5.6)		107 (72.8)	73 (33.5)	

a Two-sided Fisher’s exact tests.

b Excluded 4 patients with mixed intestinal and diffuse types in validation cohort.

### Correlation between p53 Expression and Clinicopathological Features in Patients Treated with Surgery Alone

In all three cohorts, we testified that p53 expression in cancerous tissues was significantly correlated with lymph node metastasis (N-category). Increased expression of p53 was closely associated with higher TNM stage in training and testing cohorts, but without significance in the validation cohort. Furthermore, increased expression of p53 was notably related to other clinicopathological characteristics, such as depth of invasion (T-category), distant metastasis (M-category), tumor diameter and histological type in respective cohort. Interestingly, in all patients treated with surgery alone (n = 817), we found more intestinal-type patients showed low while more diffuse-type patients showed high p53 expression (*P*<.001, data not shown). Expression of p53 had no correlation with age and gender (Table 1).

### Correlation of p53 Expression and OS in Patients Treated with Surgery Alone

**Figure 2:**
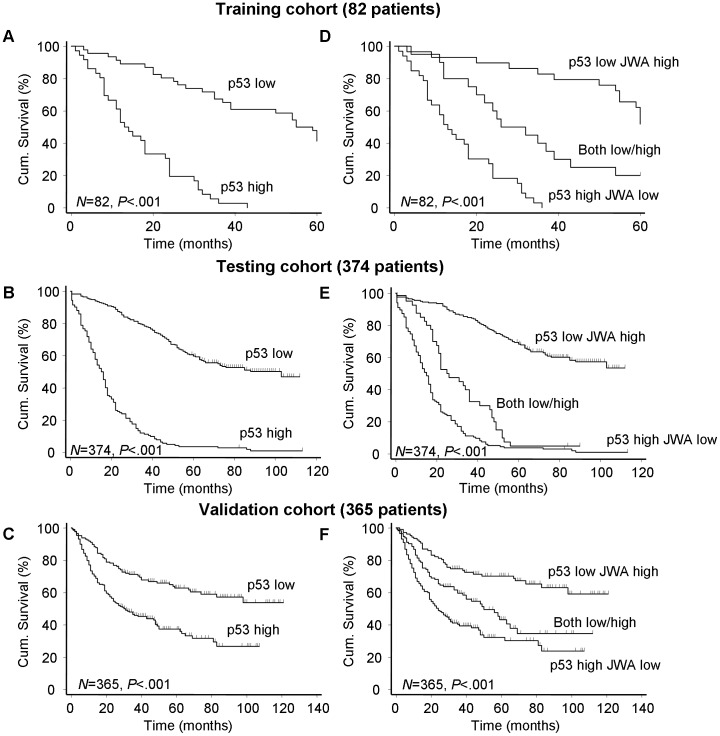
Survival curves according to expression pattern of p53 or p53/JWA in three cohorts. (A–C) Kaplan-Meier curves depicting overall survival according to expression pattern of p53. (D–F) Kaplan-Meier curves depicting overall survival according to expression pattern of p53/JWA. *P* values were calculated with the log-rank test.

In the training cohort, 82 primary tumor samples suitable for analysis showed a statistically significant negative correlation between p53 expression and overall 5-year survival using Kaplan-Meier survival curves (*P*<.001). These findings were validated in two independent and larger cohorts of gastric cancer patients with minimum five years follow-up (n = 374 and n = 365, respectively) (Fig. 2A–C). Other significant negative predictors for survival by univariate analysis in the three independent cohorts were lymph node metastasis (N-category, *P*<.01 for all, Table S2) and clinical TNM stage (*P*≤.001 for all, Table S2).

**Figure 3:**
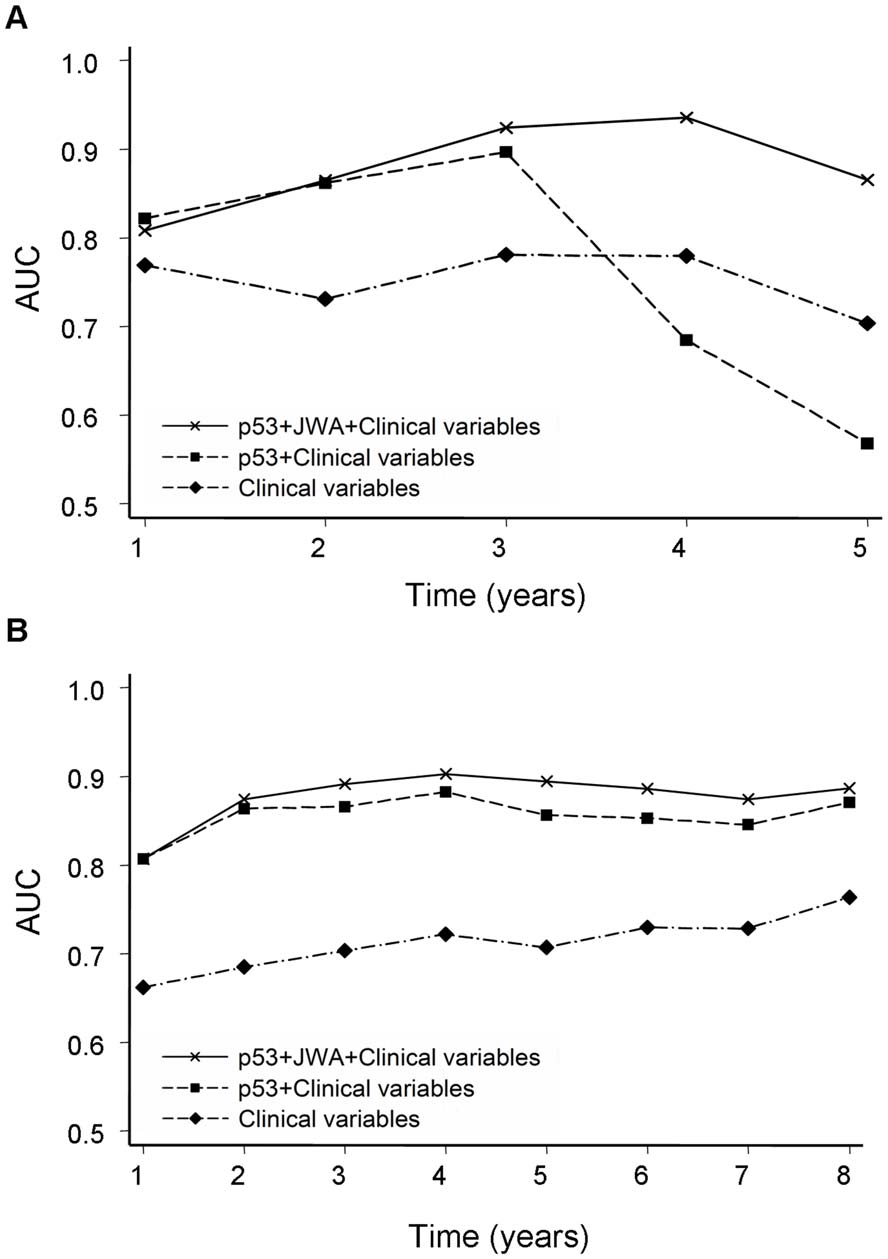
ROC analyses for clinical risk score, or the combination of p53 or p53 plus JWA. (A) Time-dependent ROC analyses in the training cohort. (B) Time-dependent ROC analyses in the testing cohort. AUC = area under the curve.

The multivariate Cox regression analysis indicated that low p53 expression was an independent positive prognostic factor for gastric cancer in all three cohorts (*P*<.001 for all, Table 2). To further evaluate the prognostic value of p53 expression, we conducted a time-dependent ROC analysis for the censored data, which indicated that combination of clinical risk score (TNM stage, histological type and tumor diameter) and p53 contributed much more than clinical parameters alone in both training and testing cohorts (Fig. 3). For example, in the testing cohort, the AUC at year 5 was 0.707 (95% CI = 0.653–0.761) for clinical risk score, whereas it was significantly increased to 0.856 (95% CI = 0.818–0.894) when combined with p53 risk score. However, this effect was not significant in the validation cohort due to the relatively higher AUC (about 0.8) of clinical predictors (Fig. S4).

**Table 2:** Multivariate Cox regression analysis of p53 or p53/JWA expression and clinicopathologic variables predicting survival in three cohorts of gastric cancers treated with surgery alone.

Variables	Training cohort(n = 82cases)		Testing cohort(n = 374cases)		Validation cohort(n = 365cases)	
	HR (95% CI)	*P* a	HR (95% CI)	*P* a	HR (95% CI)	*P* a
p53						
Age (≤65 vs. >65)	1.49 (0.75–2.95)	.256	1.16 (0.89–1.51)	.262	0.84 (0.63–1.12)	.242
Gender (male vs. female)	1.58 (0.89–2.83)	.120	1.01 (0.77–1.32)	.950	1.17 (0.83–1.64)	.365
Histological type (diffuse vs. intestinal)	1.11 (0.63–1.96)	.720	1.12 (0.86–1.44)	.397	1.53 (1.10–2.13)	.011
Tumor diameter (≤5 cm vs. >5 cm)	0.89 (0.52–1.50)	.657	1.49 (1.10–2.01)	.011	1.46 (1.08–1.97)	.014
TNM stage (I–II vs. III/IV)	1.98 (0.95–4.13)	.069	1.48 (1.05–2.07)	.023	4.15 (2.85–6.03)	<.001
p53 expression (low vs. high)	6.92 (3.50–13.68)	<.001	6.17 (4.64–8.21)	<.001	1.85 (1.34–2.56)	<.001
p53/JWA						
Age (≤65 vs. >65)	1.45 (0.73–2.87)	.290	1.19 (0.91–1.54)	.203	0.83 (0.62–1.11)	.216
Gender (male vs. female)	1.87 (1.03–3.39)	.039	0.96 (0.73–1.25)	.750	1.14 (0.81–1.61)	.439
Histological type (diffuse vs. intestinal)	1.05 (0.61–1.80)	.859	0.97 (0.75–1.25)	.819	1.46 (1.05–2.04)	.024
Tumor diameter (≤5 cm vs. >5 cm)	0.99 (0.57–1.70)	.959	1.51 (1.11–2.05)	.008	1.51 (1.12–2.03)	.007
TNM stage (I–II vs. III/IV)	0.79 (0.34–1.83)	.578	1.36 (0.97–1.91)	.076	3.97 (2.72–5.79)	<.001
p53/JWA expression(p53 high JWA low vs. both low/high )	0.24 (0.11–0.49)	<.001	0.57 (0.40–0.83)	.003	0.79 (0.57–1.11)	.176
(p53 high JWA low vs. p53 low JWA high)	0.05 (0.02–0.14)	<.001	0.12 (0.09–0.16)	<.001	0.45 (0.30–0.67)	<.001

a Multivariate Cox regression analysis including age, gender, TNM stage, tumor diameter, histological type, p53 or p53/JWA proteins expression status.

Abbreviations: HR: hazard ratio; CI: confidence interval.

### Correlation between p53 Expression and OS in Patients with Adjuvant Chemotherapy

**Figure 4:**
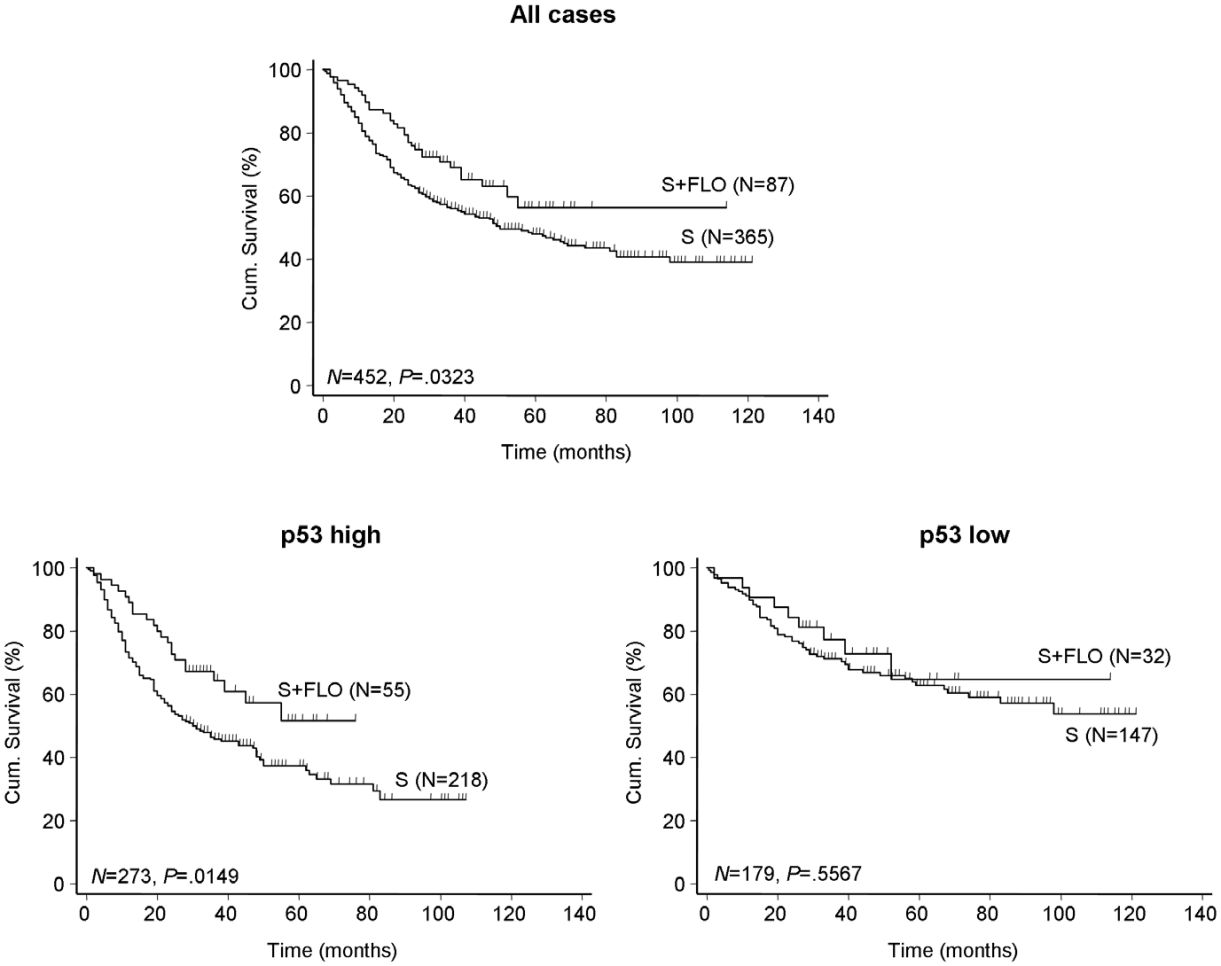
Survival curves according to p53 expression in the validation cohort treated with or without FLO. *P* values were calculated with the log-rank test. Note: S, surgery alone; FLO, fluorouracil-leucovorin-oxaliplatin.

In testing and validation cohorts, OS was analyzed between the patients who received adjuvant chemotherapy versus those who did not. Data showed no difference in OS between surgery only group and any regimen of postoperative adjuvant chemotherapy (data not shown) except in the group receiving fluorouracil- leucovorin-oxaliplatin (FLO) (n = 87, log-rank test, *P* = .032, Fig. 4). A multivariate Cox regression analysis including six variables (age, gender, TNM stage, histological types, tumor diameter and chemotherapy) was performed to indicate the benefit of chemotherapy on OS. There was a statistically significant benefit for those who received FLO chemotherapy after operation over the surgery alone group (HR = 0.55; 95% CI = 0.37–0.82, data not shown). Notably, this effect was only found in patients with high p53 expression where adjuvant FLO obviously increased OS as compared with surgery alone (HR = 0.56; 95% CI = 0.35–0.89, Table S3; Log-rank test, *P* = .014, Fig. 4).

We also analyzed the significance of another platinum-based chemotherapy, fluorouracil-leucovorin-platinol (FLP) regimen (n = 79) in resectable gastric cancer. The results did not present a significant survival difference (log-rank test, *P* = .134, Fig. S5), whereas patients with low p53 expression receiving FLP regimen even displayed a worse survival compared with those receiving surgery only (*P* = .003, Fig. S5). Moreover, patients undergoing FLP treatment with high p53 expression had no significant survival discrepancy compared to those with surgery alone (*P* = .648, Fig. S5). Further multivariate analysis elucidated that higher risk for mortality was observed in patients with low p53 expression receiving FLP treatment compared with surgery only (HR = 1.91, 95% CI = 1.08 to 3.36, Table S4).

### Synergetic Effect of p53 with JWA Expression on OS in Patients Treated with Surgery only or Adjuvant Chemotherapy

The patients with surgery alone were further stratified into three distinct groups on the basis of staining for p53 and JWA: p53 high with JWA low, p53 low with JWA high and both high or both low. It was shown that patients with p53 low and JWA high had a best outcome of survival in these three groups (*P*<.001, log-rank test; Fig. 2D–F). The multivariate Cox regression analysis demonstrated that combined expression of low p53 and high JWA was independent positive prognostic factor for gastric cancer in all three cohorts (*P*<.001 for all, Table 2). To further evaluate the prognostic value of p53 plus JWA expression, we conducted another time-dependent ROC analysis, which indicated that the combination of the clinical risk score and p53 plus JWA contributed more than the cooperation of clinical risk score with only p53 in both of training and testing cohorts (Fig. 3). For instance, in the training cohort, the AUC at year 5 was 0.568 (95% CI = 0.400–0.737) for clinical risk score plus p53 risk score, whereas it was significantly increased to 0.865 (95% CI = 0.786–0.944) when combination of the clinical risk score with p53 plus JWA risk score was used. However, this effect was again not apparent in the validation cohort due to the reason mentioned above (Fig. S4).

Based on contribution of p53 expression level to chemotherapy outcome, we stratified such patients in coordination with JWA as follows: FLO treatment group with high p53 expression was divided into p53 high with JWA low and p53 high with JWA high; and FLP treatment group with low p53 expression was also classified into p53 low with JWA low and p53 low with JWA high. OS was reevaluated between these subgroups. However, no evident survival disparity was presented in synergic pattern with JWA (Fig. S6).

## Discussion

Gastric cancer is a heterogeneous disease where the outcome varies even in patients with similar clinical and pathological features. Even with surgery in early stages, the prognosis may be dismal, and adjuvant chemotherapy is effective only in subgroups of patients. New molecular markers are needed as traditional staging systems for gastric cancer are insufficient for predicting outcomes [24]. In this study, we recorded and demonstrated that high expression of p53 was significantly correlated with unfavorable clinicopathologic parameters and decreased overall patient survival. Furthermore, patients with high p53 expression in tumors acquired remarkable survival benefit from adjuvant first-line platinum-based chemotherapy (FLO or FLP). The synergy between p53 and JWA in predicting patient outcome was demonstrated, while no obvious elevated predictive value concerning chemotherapy was observed.

In the present study, we found evidently increased expression of p53 protein in gastric cancer tissues versus matched normal mucosa, suggesting a potentially important status of p53 in gastric carcinogenesis. Reportedly, the *p53* gene is mutated in nearly 50% of all human tumors including gastric cancer [25] and the mutated protein remains within the cells for a longer time, allowing detection by immunohistochemistry [26], [27]. Therefore, it is generally accepted that positive staining mainly represents the mutant forms of p53, whereas wild-type p53 protein is degraded more rapidly and appears weak or negative staining [10], [26]. Our data were consistent with studies from other investigators, which revealed that positive p53 expression was associated with unfavorable clinicopathologic characteristics and a poor prognosis of gastric cancer patients who have undergone curative gastrectomy [28]–[30]. It’s worth noting that some differences in clinicopathologic features exist between Nantong and Yixing cohorts. Since Nantong Cancer Hospital is a cancer special hospital while Yixing People’s Hospital is a comprehensive one, patients in the former are often at more advanced stage compared to the latter. Accordingly, patients in Nantong cohort showed worse clinicopathologic characteristics. Additionally, p53 and JWA expression were only detected in tissues here but not in blood or serum of gastric cancer patients, due to they are not secreted proteins. Interestingly, a previous study showed a strong correlation between high p53 auto-antibodies in serum of gastric adenocarcinoma patients and poor prognosis, lymph node metastasis and low differentiation [31]. Moreover, it was shown that p53 expression in gastric cancer tissues correlated with serum p53 antibodies [32]. We did not have sera collected for a similar analysis, but this would be very interesting as a blood test is simpler and probably more reproducible than immonohistochemistry. As for JWA, a serum test is still pending and needs to be demonstrated in the future.

A fascinating and important question attracting us most is why could high expression of p53 play precisely opposite roles on prognostic and predictive effect. This phenomenon could be attributed to different forms of p53 and their distinct properties in cancer biology and treatment resistance. It is widely known mutant p53 proteins are highly expressed in many cancers and contribute to malignant transformation, proliferation, and metastasis in part by inhibiting wild-type p53 as well as other members of p53 family [6], [33]. Mouse models bearing knock-in mutations of p53 showed that mutant p53 proteins can drive tumor formation, invasion and metastasis through dominant negative inhibition of wild-type p53 along with gain-of-function or ‘neomorphic’ activities that can inhibit or activate the function of other proteins [34], [35]. Likewise in our study, high p53 expression in gastric tumors correlated with lymph node metastasis, higher TNM stage and other unfavorable clinicopathologic features, leading to a more aggressive phenotype with a worse prognosis when untreated. In contrast, the positive predictive effect of high p53 expression on survival in both platinum treated patients may point to a dual role of p53 in chemo-resistance. Currently, a major obstacle in platinum chemotherapy is the repair of platinum-damaged DNA that results in increased resistance, reduced apoptosis, and finally treatment failure. Wild-type p53 may partially play such a role during this process via cell cycle arrest and following repair of damage induced by chemical agents [36]–[39]. Conversely, mutant p53 proteins are unable to function in DNA damage repair as their wild-type counterpart where the patients become more sensitive to chemicals [36], [40], [41]. However, the detailed mechanism of mutant p53 in platinum treatment needs to be further investigated. It must be noted that the patients received more survival benefit from FLO than from FLP, which may be partially due to that FLO reduces toxicity as compared with FLP as noted before [19].

JWA is a typical stress response gene as well as a tumor suppressor [18], [42]. In vitro and in vivo studies confirmed that loss of JWA suppressed cell differentiation and increased cell migration and metastasis [42]–[44]. Our latest results manifested low JWA expression in gastric tumors correlated with unfavorable clinicopathologic indicators but better chemotherapy outcome [19]. In the present study, we found that JWA improved the prognostic value of p53, suggesting that loss of JWA combined with p53 mutation may increase tumor aggressiveness and metastasis, which probably because JWA is also a member of DNA repair pathway and possibly play a mutual role in gastric carcinogenesis. Intriguingly, even if both markers separately were predictive for platinum treatment, the combination did not improve the predictive value, but this result is hampered by small group of patients in the subgroups. A larger number of patients receiving platinum-based chemotherapy regimens should be included in our future study to further validate our results. Furthermore, no direct regulatory effect was demonstrated between p53 and JWA (data not shown), perhaps they act in different repair pathways [17], [18], [45]–[48] and indirect relationship exists between them under certain situations. Moreover, p53 and JWA may synergistically regulate the same molecules or signal pathways which needs to be elucidated in further studies.

In summary, we revealed a combined value of tumoral p53 with JWA as efficient prognostic factors for the first time, to the best of our knowledge. Although a definite co-action of these two proteins is yet to be proven, it may provide the potential predictors for adjuvant chemotherapy with a platinum-based regimen.

## Supporting Information

Figure S1 Consort diagram for the tissue microarray study.
(TIF)

Figure S2 Representative images of p53 immunohistochemical staining in human gastric cancer. A, negative staining; B, weak positive staining; C, moderate positive staining; D, strong positive staining (A–D: scale bar, 25 µm).
(TIF)

Figure S3 ROC curves depicting the relation between area under the curve (AUC) at different cutoff values of p53 immunoreactivity score (IRS) for 1, 3 and 5 years of overall survival time.
(TIF)

Figure S4 ROC analyses for the clinical risk score, the combined p53 or p53 plus JWA and clinical risk score in the validation cohort. AUC = area under the curve.
(TIF)

Figure S5 Survival curves according to p53 expression pattern in validation cohort treated with or without FLP. *P* values were calculated with the log-rank test. Note: S, surgery alone; FLP, fluorouracil-leucovorin-platinol.
(TIF)

Figure S6 Survival curves depicting synergetic effect of p53/JWA expression pattern in validation cohort receiving adjuvant chemotherapy. *P* values were calculated with the log-rank test. Note: S, surgery alone; FLO, fluorouracil- leucovorin-oxaliplatin; FLP, fluorouracil-leucovorin-platinol.
(TIF)

Table S1 The distributions of demographic and clinicopathologic characteristics of patients treated with or without chemotherapy.
(DOC)

Table S2 Univariate Cox regression analysis of p53 or p53/JWA expression and clinicopathologic variables predicting survival in three cohorts of gastric cancers treated with surgery alone.
(DOC)

Table S3 Multivariate Cox regression analysis assessing the predictive significance of p53 expression in radical gastrectomy patients treated with or without FLO.
(DOC)

Table S4 Multivariate Cox regression analysis assessing the predictive significance of p53 expression in radical gastrectomy patients treated with or without FLP.
(DOC)
