# Supplementary figures and images for: Synergistic Role between p53 and JWA: Prognostic and Predictive Biomarkers in Gastric Cancer
Source: PLoS One. 2012 Dec 21;7(12):e52348. doi: 10.1371/journal.pone.0052348 (PMC3528747; doi:10.1371/journal.pone.0052348)

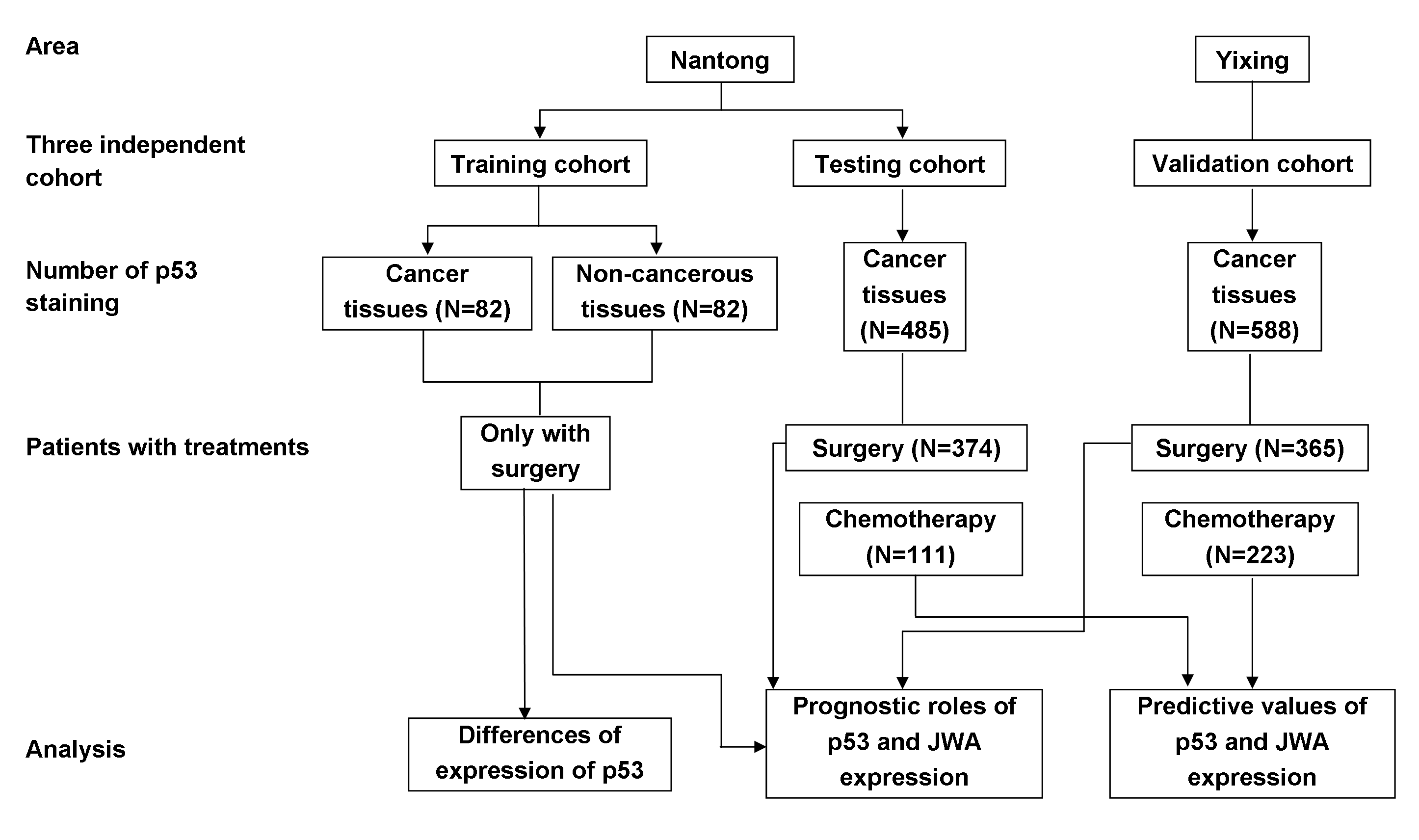

Supplement: Figure S1 — Consort diagram for the tissue microarray study. (TIF) [file pone.0052348.s001.tif]

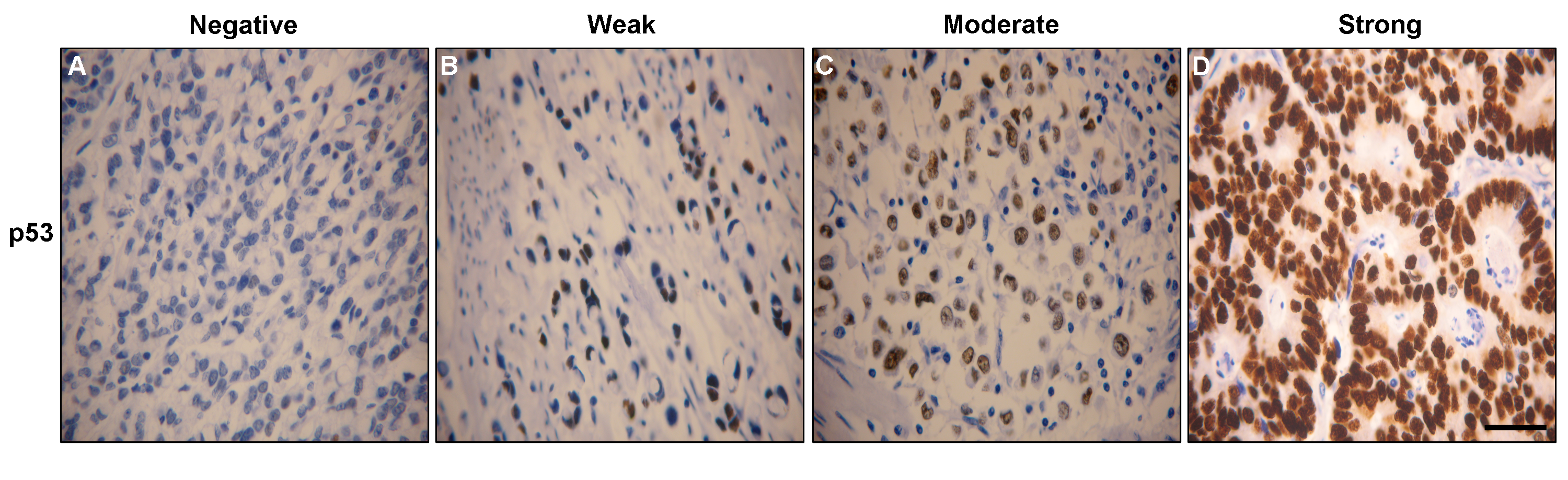

Supplement: Figure S2 — Representative images of p53 immunohistochemical staining in human gastric cancer. A, negative staining; B, weak positive staining; C, moderate positive staining; D, strong positive staining (A–D: scale bar, 25 µm). (TIF) [file pone.0052348.s002.tif]

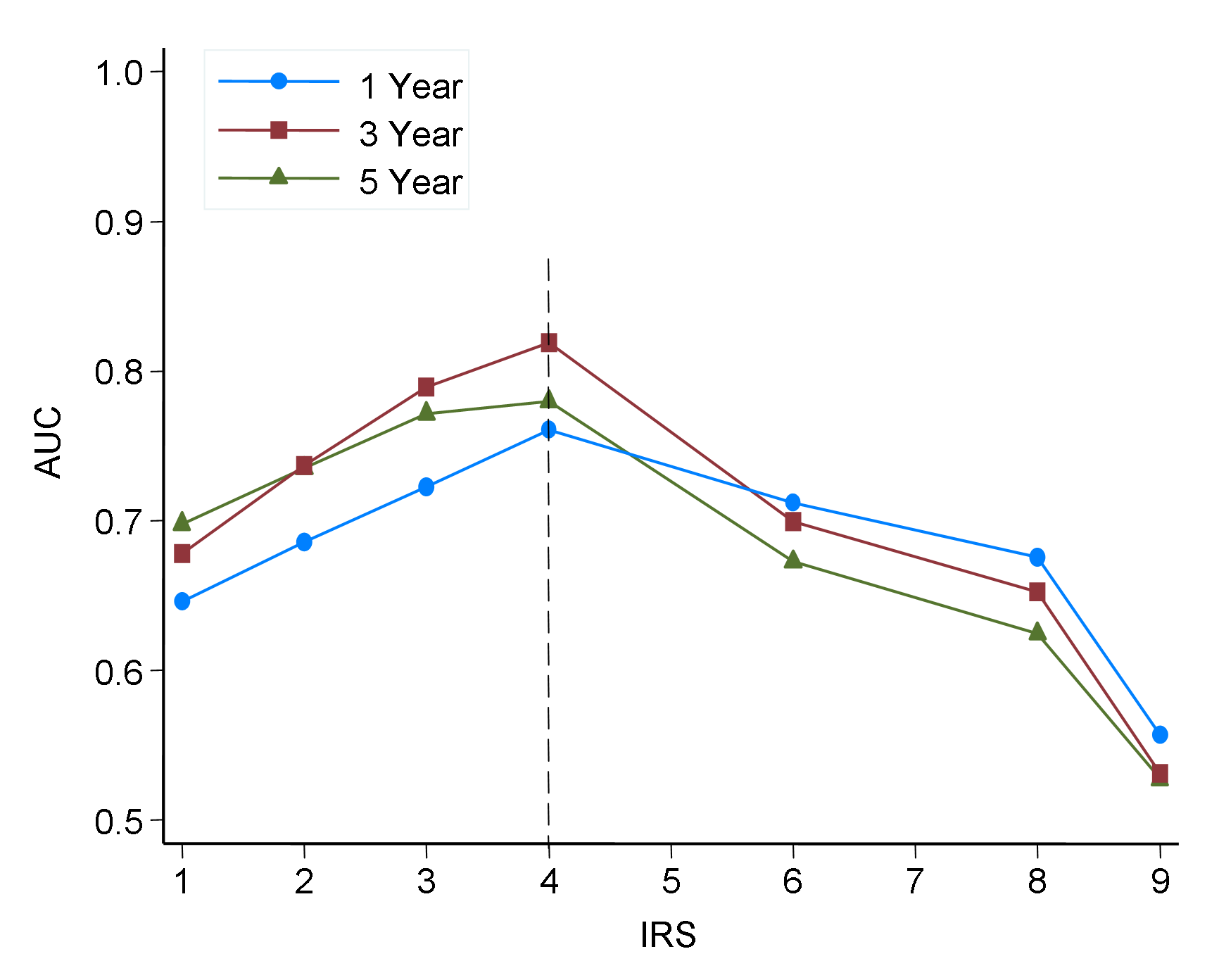

Supplement: Figure S3 — ROC curves depicting the relation between area under the curve (AUC) at different cutoff values of p53 immunoreactivity score (IRS) for 1, 3 and 5 years of overall survival time. (TIF) [file pone.0052348.s003.tif]

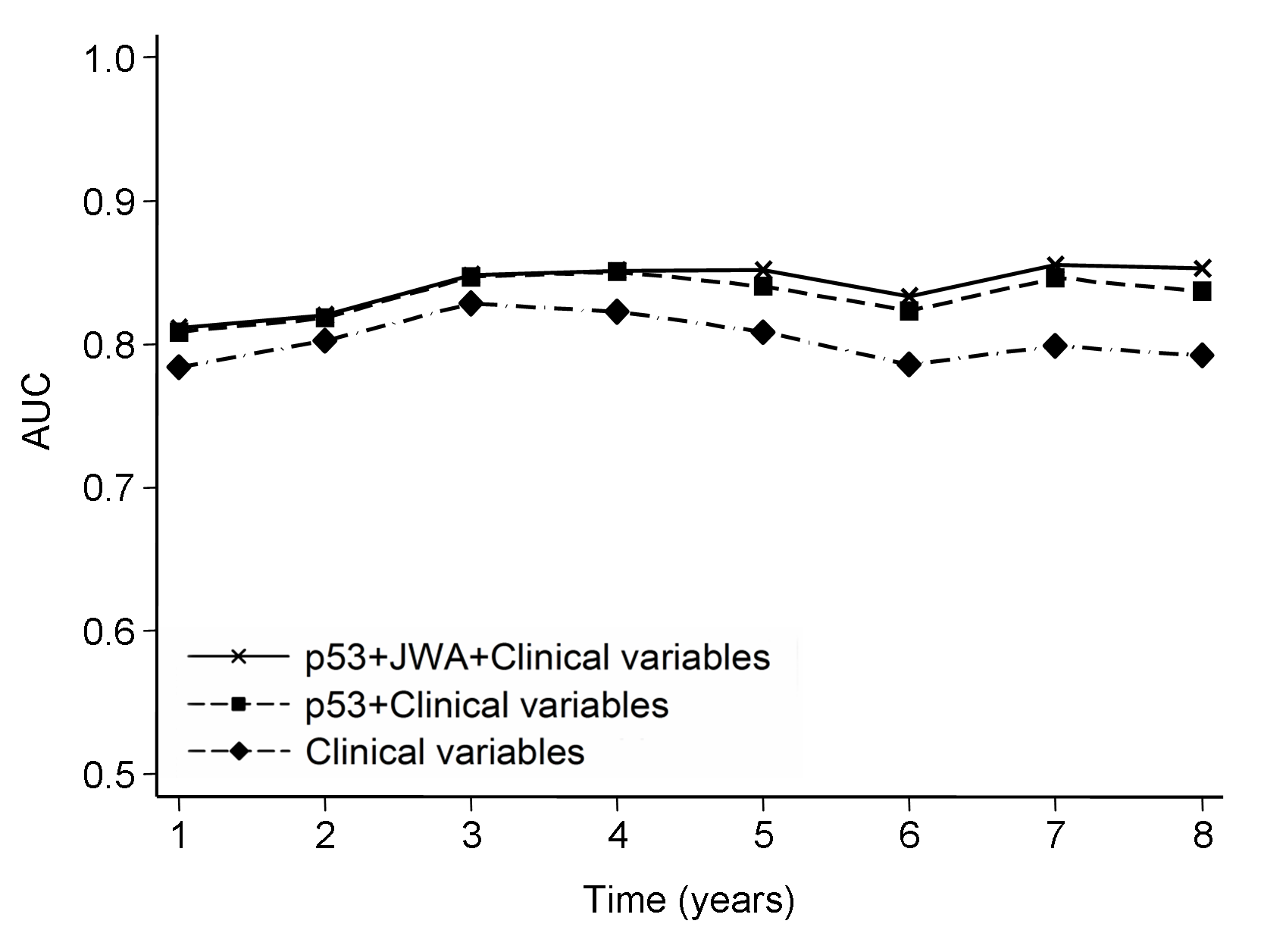

Supplement: Figure S4 — ROC analyses for the clinical risk score, the combined p53 or p53 plus JWA and clinical risk score in the validation cohort. AUC = area under the curve. (TIF) [file pone.0052348.s004.tif]

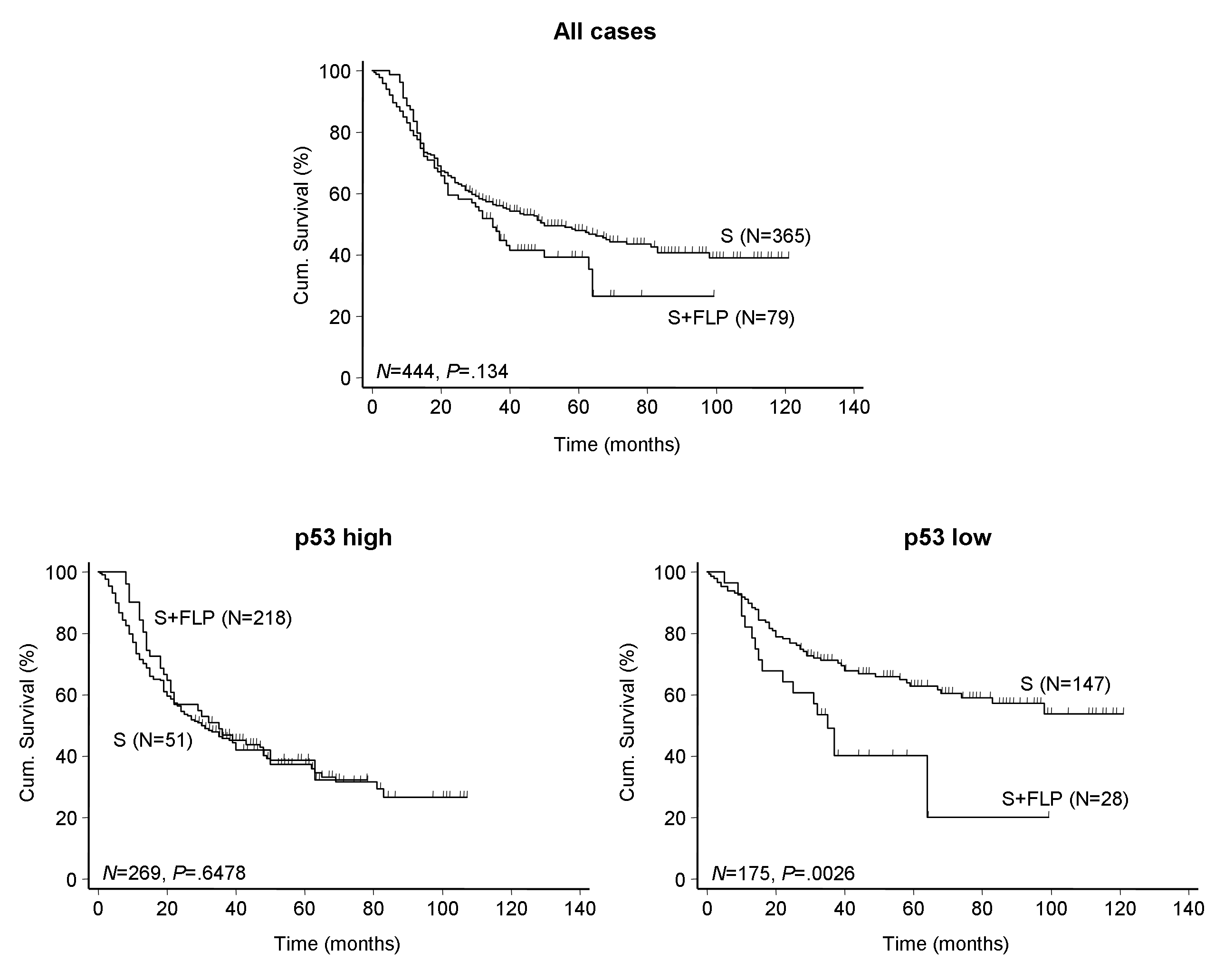

Supplement: Figure S5 — Survival curves according to p53 expression pattern in validation cohort treated with or without FLP. P values were calculated with the log-rank test. Note: S, surgery alone; FLP, fluorouracil-leucovorin-platinol. (TIF) [file pone.0052348.s005.tif]

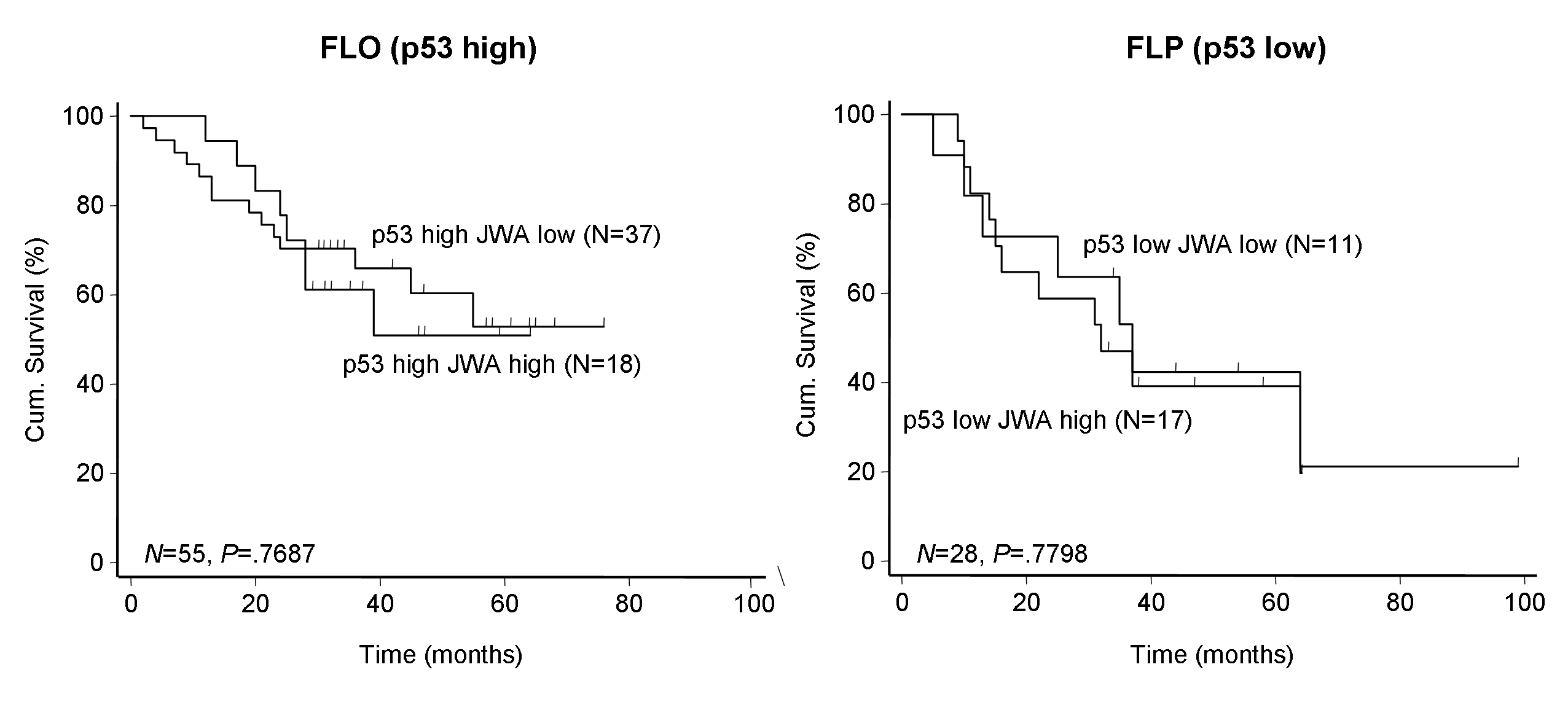

Supplement: Figure S6 — Survival curves depicting synergetic effect of p53/JWA expression pattern in validation cohort receiving adjuvant chemotherapy. P values were calculated with the log-rank test. Note: S, surgery alone; FLO, fluorouracil- leucovorin-oxaliplatin; FLP, fluorouracil-leucovorin-platinol. (TIF) [file pone.0052348.s006.tif]
