# Supplementary material for: Synergistic Role between p53 and JWA: Prognostic and Predictive Biomarkers in Gastric Cancer
Source: PLoS One. 2012 Dec 21;7(12):e52348. doi: 10.1371/journal.pone.0052348 (PMC3528747; doi:10.1371/journal.pone.0052348)
Supplement: Table S1 — The distributions of demographic and clinicopathologic characteristics of patients treated with or without chemotherapy. (DOC) [file pone.0052348.s007.doc]

**Table S1.** The distributions of demographic and clinicopathologic characteristics of patients treated with or without chemotherapy.

| **Variables** | **only with surgery N=365** | **surgery-FLO**  **N=87** | **surgery-FLP**  **N=79** | ***p^a^*** | ***p^b^*** | ***p^c^*** |
| --- | --- | --- | --- | --- | --- | --- |
| All patients | 365 | 87 | 79 |  |  |  |
| Age (years) |  |  |  |  |  |  |
| ≤65 | 160 | 51 | 45 | .017 | .035 | .876 |
| >65 | 205 | 36 | 34 |  |  |  |
| Gender |  |  |  |  |  |  |
| Males | 282 | 72 | 64 | .312 | .550 | .841 |
| Females | 83 | 15 | 15 |  |  |  |
| Depth of invasion |  |  |  |  |  |  |
| T1/T2 | 126 | 15 | 13 | .002 | .001 | 1.000 |
| T3/T4 | 239 | 72 | 66 |  |  |  |
| Lymph node metastasis |  |  |  |  |  |  |
| N0 | 139 | 27 | 18 | .265 | .010 | .294 |
| N1/N2/N3 | 226 | 60 | 61 |  |  |  |
| Distant metastasis |  |  |  |  |  |  |
| M0 | 351 | 81 | 74 | .243 | .354 | 1.000 |
| M1 | 14 | 6 | 5 |  |  |  |
| TNM stage |  |  |  |  |  |  |
| I | 93 | 7 | 7 | .001 | .004 | .667 |
| II | 81 | 25 | 17 |  |  |  |
| III | 181 | 54 | 53 |  |  |  |
| IV | 10 | 1 | 2 |  |  |  |
| Tumor diameter |  |  |  |  |  |  |
| ≤5cm | 207 | 48 | 44 | .811 | .901 | 1.000 |
| >5cm | 158 | 39 | 35 |  |  |  |
| Histological type^d^ |  |  |  |  |  |  |
| Intestinal | 150 | 53 | 27 | .001 | .255 | .001 |
| Diffuse | 211 | 34 | 52 |  |  |  |

^a^*P* values of two-sided Fisher’s exact tests for the differences between patients treated with surgery only and those with surgery-FLO.

^b^*P* values of two-sided Fisher’s exact tests for the differences between patients treated with surgery only and those with surgery-FLP.

^c^*P* values of two-sided Fisher’s exact tests for the differences between patients treated with surgery-FLO and those with surgery-FLP.

^d^Excluded 4 patients with mixed intestinal and diffuse types in patients only with surgery.

Abbreviations: FLO, fluorouracil-leucovorin-oxaliplatin; FLP, fluorouracil- leucovorin- platinol.
