# Supplementary material for: Synergistic Role between p53 and JWA: Prognostic and Predictive Biomarkers in Gastric Cancer
Source: PLoS One. 2012 Dec 21;7(12):e52348. doi: 10.1371/journal.pone.0052348 (PMC3528747; doi:10.1371/journal.pone.0052348)
Supplement: Table S2 — Univariate Cox regression analysis of p53 or p53/JWA expression and clinicopathologic variables predicting survival in three cohorts of gastric cancers treated with surgery alone. (DOC) [file pone.0052348.s008.doc]

Table S2. univariate Cox regression analysis of p53 or p53/JWA expression and clinicopathologic variables predicting survival in three cohorts of gastric cancers treated with surgery alone.

| Variables | Training cohort (n=82 cases) | | Testing cohort (n=374 cases) | | Validation cohort (n=365 cases) | |
| --- | --- | --- | --- | --- | --- | --- |
|  | HR (95% CI) | *P* | HR (95% CI) | *P* | HR (95% CI) | *P* |
| Age (≤65 vs. >65) | 0.88 (0.48-1.63) | .692 | 1.05 (0.81-1.35) | .732 | 0.76 (0.57-1.01) | .058 |
| Gender (male vs. female) | 1.40 (0.81-2.42) | .227 | 1.10 (0.84-1.42) | .524 | 1.10 (0.79-1.55) | .568 |
| Depth of invasion ( T1/T2 vs. T3/T4) | 1.59 (0.50-5.08) | .434 | 2.48 (1.69-3.63) | < .001 | 5.56 (3.59-8.62) | < .001 |
| Lymph node metastasis (N0 vs. N1/N2/N3) | 2.52 (1.34-4.77) | .004 | 2.75 (1.98-3.82) | < .001 | 5.11 (3.43-7.60) | < .001 |
| Distant metastasis (M0 vs. M1) | 2.93(1.59-5.38) | .001 | 1.53(0.89-2.62) | .124 | 2.79(1.55-5.02) | .001 |
| TNM stage (I-II vs. III-IV) | 2.74 (1.54-4.87) | .001 | 2.46 (1.82-3.31) | < .001 | 5.45 (3.83-7.75) | < .001 |
| Tumor diameter (≤5cm vs. >5cm) | 1.17 (0.71-1.91) | .543 | 2.32 (1.76-3.05) | < .001 | 2.28 (1.71-3.04) | < .001 |
| Histological type (diffused vs. intestinal) | 1.20 (0.73-1.96) | .478 | 1.51 (1.18-1.94) | .001 | 2.27 (1.64-3.12) | < .001 |
| p53 expression (low vs. high) | 7.25 (3.89-13.50) | < .001 | 7.36 (5.58-9.71) | < .001 | 2.22 (1.62-3.05) | < .001 |
| p53/JWA expression  (p53 high JWA low vs. both low/high) | 0.27 (0.14-0.55) | < .001 | 0.52 (0.36-0.74) | < .001 | 0.63 (0.46-0.88) | .006 |
| (p53 high JWA low vs. p53 low JWA high) | 0.05 (0.02-0.15) | < .001 | 0.11 (0.08-0.14) | < .001 | 0.32 (0.22-0.49) | < .001 |

Abbreviations: HR: hazard ratio; CI: confidence interval.
