# Supplementary material for: Synergistic Role between p53 and JWA: Prognostic and Predictive Biomarkers in Gastric Cancer
Source: PLoS One. 2012 Dec 21;7(12):e52348. doi: 10.1371/journal.pone.0052348 (PMC3528747; doi:10.1371/journal.pone.0052348)
Supplement: Table S3 — Multivariate Cox regression analysis assessing the predictive significance of p53 expression in radical gastrectomy patients treated with or without FLO. (DOC) [file pone.0052348.s009.doc]

**Table S3.** Multivariate Cox regression analysis assessing the predictive significance of p53 expression in radical gastrectomy patients treated with or without FLO.

| Variables^a^ | p53 expression ( n=452) | | | |
| --- | --- | --- | --- | --- |
|  | Low (n=179) | | High (n=273) | |
|  | HR (95% CI) | *p*^a^ | HR (95% CI) | *p*^a^ |
| Surgery | 1.00 |  | 1.00 |  |
| Surgery-FLO | 0.50 (0.24-1.04) | .062 | 0.56 (0.35-0.89) | .014 |

^a^Multivariate Cox regression analysis was performed with six variables (age, gender, TNM stage, histological type, tumor diameter, FLO treatment).

Abbreviations: FLO: fluorouracil-leucovorin-oxaliplatin; HR: hazard ratio; CI: confidence interval.
