# Supplementary material for: Synergistic Role between p53 and JWA: Prognostic and Predictive Biomarkers in Gastric Cancer
Source: PLoS One. 2012 Dec 21;7(12):e52348. doi: 10.1371/journal.pone.0052348 (PMC3528747; doi:10.1371/journal.pone.0052348)
Supplement: Table S4 — Multivariate Cox regression analysis assessing the predictive significance of p53 expression in radical gastrectomy patients treated with or without FLP. (DOC) [file pone.0052348.s010.doc]

**Table S4.** Multivariate Cox regression analysis assessing the predictive significance of p53 expression in radical gastrectomy patients treated with or without FLP.

| Variables^a^ | p53 expression ( n=444) | | | |
| --- | --- | --- | --- | --- |
|  | Low (n=175) | | High (n=269) | |
|  | HR (95% CI) | *p*^a^ | HR (95% CI) | *p*^a^ |
| Surgery | 1.00 |  | 1.00 |  |
| Surgery-FLP | 1.91 (1.08-3.36) | .025 | 0.70 (0.47-1.04) | .079 |

^a^Multivariate Cox regression analysis was performed with six variables (age, gender, TNM stage, histological type, tumor diameter, FLP treatment).

Abbreviations: FLP: fluorouracil-leucovorin- platinol; HR: hazard ratio; CI: confidence interval.
